# The RNA-Seq data analysis shows how the ontogenesis defines aging

**DOI:** 10.3389/fragi.2023.1143334

**Published:** 2023-03-14

**Authors:** Lev Salnikov, Saveli Goldberg, Heena Rijhwani, Yuran Shi, Eugene Pinsky

**Affiliations:** ^1^ AntiCA Biomed, San Diego, CA, United States; ^2^ Department of Radiation Oncology, Mass General Hospital, Boston, MA, United Kingdom; ^3^ Department of Computer Science, Met College, Boston University, Boston, MA, United Kingdom; ^4^ Department of Computer Science, Brandeis University, Waltham, MA, United Kingdom

**Keywords:** aging, ontogenesis, housekeeping genes, integrative genes, RNA-seq data analysis

## Abstract

This paper presents a global statistical analysis of the RNA-Seq results of the entire *Mus musculus* genome. We explain aging by a gradual redistribution of limited resources between two major tasks of the organism: its self-sustenance based on the function of the housekeeping gene group (HG) and functional differentiation provided by the integrative gene group (IntG). All known disorders associated with aging are the result of a deficiency in the repair processes provided by the cellular infrastructure. Understanding exactly how this deficiency arises is our primary goal. Analysis of RNA production data of 35,630 genes, from which 5,101 were identified as HG genes, showed that RNA production levels in the HG and IntG genes had statistically significant differences (*p*-value <0.0001) throughout the entire observation period. In the reproductive period of life, which has the lowest actual mortality risk for *Mus musculus*, changes in the age dynamics of RNA production occur. The statistically significant dynamics of the decrease of RNA production in the HG group in contrast to the IntG group was determined (*p*-value = 0.0045). The trend toward significant shift in the HG/IntG ratio occurs after the end of the reproductive period, coinciding with the beginning of the mortality rate increase in *Mus musculus* indirectly supports our hypothesis. The results demonstrate a different orientation of the impact of ontogenesis regulatory mechanisms on the groups of genes representing cell infrastructures and their organismal functions, making the chosen direction promising for further research and understanding the mechanisms of aging.

## Introduction

The biological mechanism of aging remains one of the main mysteries in modern biology. Most scientists dealing with this problem agree in the opinion that aging is either the result of a purposeful evolutionary program inherent in the genome (Larocca et al., 2021; De Magalhães Church., 2005; Bilinski et al., 2021), or a consequence of spontaneous disturbances in the organism leading to maladaptation (Soto-Palma et al., 2022; Kinzina et al., 2019). At the same time both approaches to the causes of aging are not yet presented by the description of a specific mechanism explaining the emergence and acceleration of aging after reaching fertility (Walker, 2022; West et al., 2019).

In our work we present the analysis of RNA-Sequencing (RNA-Seq) results from the point of view of the theoretical model we presented earlier, where the inseparable connection between the processes of aging and ontogenesis is obvious (Salnikov and Baramiya, 2020; Salnikov and Baramiya, 2021). The definition of aging itself as a universal process is given in (Gavrilov Gavrilova, 2001), where it is shown that this phenomenon will occur in any objects where insufficient recovery of damaged or consumed resources occurs. In the case of a multicellular organism, its aging is also caused by a decrease in resources required by its cells for their reparation and tissue regeneration and starts at the moment when its recovery begins to be incomplete (Naviaux, 2019).

At the end of the 19th century, the famous biologist August Weissmann presented his theory of germplasm, where he described the division of roles between cells, making possible the evolution of complex multicellular life forms (Weissman, 1891). To make the transition from unicellular to multicellular organisms, two fundamentally different types of cells originally constituting a multicellular organism emerged in the course of evolution, these are the mortal Soma or body cells, which eventually age and die, and the immortal self-renewing germline cells, which carry hereditary information for the next-generation. It is the ability of germline cells to self-renew that makes them immortal.

In our theoretical model we adhere to a partially similar but different basic distinction, which is based on the concept of functional division of the metazoa cell genome presented in our publications (Salnikov Baramiya, 2020; Salnikov Baramiya, 2021). We argue that during ontogenesis (reflecting phylogeny or evolutionary origin), any multicellular organism is built on the basis of not all but only a part of its genome, which has changed in the course of evolution. The other part of the genome remains virtually unchanged, constantly ensuring the viability of the cell. These are the genes that are close or almost identical to those of unicellular organisms. We argue that a prerequisite for the development of an organism is the presence of two functionally independent parts in the DNA of all its cells. One of them is the most conserved part of the genome, which provides internal needs of any cell, or *cellular infrastructure*, are housekeeping genes (HG). This group of genes is essentially analogous to Weisman’s “germline” cells. The group of genes with this name was defined quite a long time ago and one of the main criteria of this group was a relatively constant level of activity of the genes included in it (Eisenberg Levanon, 2013; Wei and Ma, 2017). Another functional part of the cellular genome, are the genes providing the integrative function, or genes responsible for all specialized structures produced by cells during differentiation and creating the organism as an integrated whole (IntG). This group of genes is similar in its function to the Weisman cell line “Soma”. Confirmation of reliable differences between the functional groups we identified was presented in our previous work on genome methylation (Salnikov et al., 2022).

An essential condition for the development of any multicellular organism is the presence of the developmental program in its genome, which determines the entire course of ontogenesis. We understand the ontogenesis program as a strictly defined sequence of IntG gene expression with the assignment of an advantage in the consumption of cellular resources in order to form the organism and achieve its maximum development by the beginning of reproductive age. In our views we proceed from two basic statements: resource capabilities of any cell are always limited; the main goal of the ontogenesis program is to achieve the maximum competitive advantage by the time the organism reaches reproductive age, because natural selection is aimed at this very stage of development (Gems et al., 2021; Bilinski et al., 2021; Gems et al., 2021; Kirkwood and Holliday, 1979). This advantage, gained at a critical moment for the continuation of the species, is paid for by the suppression of autonomous and regenerative potential in the future. This situation is essentially a pleiotropic relationship (Williams,1957), but applies not to individual genes, but to the HG and IntG groups we have identified.

Note that the difference in approach between the “hyperfunction theory” (Blagosklonny, 2007; Blagosklonny, 2022; Gems, 2022) and our theoretical model is that the leading role in aging processes for us is not a direct and continuous gain of functions in the organism, or the work of the “quasiprogram”, but the decrease in activity of *cell infrastructure* leading to shift of cell resources to IntG part of the genome. In addition, the assumption of increased activity of all genes in the organism is not experimentally confirmed. On the contrary, according to the latest data, the level of metabolism providing the organism functions remains constant in people between 20 and 60 years of age (Rhoads and Anderson, 2021), which cannot provide the continuous increase of functions.

We are most interested in the period of the first third of life, during which the processes that trigger aging take place, allowing us to analyze its causes rather than consequences. From our point of view, the deficiency of reparative capabilities arising in the course of the organism development explains all aging manifestations, starting from genes DNA damage and finishing with inherent functional disorders and diseases (Walker, 2022). Actual mortality risk curve are equally applicable both for humans and laboratory animals, allows to visualize the specifics of the time interval of interest. Widely used at present statistical index of mortality probability is also a universal parameter of general resistance to external conditions. The averaged curve of *Mus musculus* mortality probability presented in (Gavrilova and Gavrilov, 2014; Hughes and Hekimi, 2016) shows a similar increase in the mortality rate depending on age. Comparing the age of humans and *Mus musculus*, we obtain an approximate correspondence in which the age of mice at 1 month corresponds to the age of humans at 3 years, 3 months correspond to 10 years, 6 months correspond to 20, 9 months–30, 12 months to 40, 15 months–50, 18 months–60, 21 months to 70, 24 months–80, and 27 correspond–90 years. We identified the following periods of ontogenesis or *Stages* for *Mus musculus*: *Stage I*—childhood and development (completion of formation of the skeletal and cardiovascular systems)—1–6 months (Maupin and Childress*.*(2019); *Stage II*—the beginning and end of the reproductive period—6–15 months; *Stage III* - the postreproductive period, 15–21 months; *Stage IV*—old age, 21–27 months. To determine the point of maximum physiological maturity we used the information provided by the growth model (West et al.,2001; Bueno Juan and Ángel, 2014). The model shows that the mass of the body first increases rapidly, and then stops its growth at a level determined by the competition between the processes of metabolism and regeneration. Using this approach, we identified the age of *Mus musculus* at 9 months as the point at which their physical growth is completed and their metabolic needs virtually disappear. It is possible to designate this age as the point of reaching the *physiological peak of development*. It follows from these data that the mortality rate itself becomes significant after the end of the reproductive period (after reaching the age of 40–50 years for humans) and respectively about 15 months for *Mus musculus* (Brust et al., 2015; Kinzina et al., 2019). For us it is the reproductive age period when the organism demonstrates the greatest resistance to external influences that is of primary interest. At this time there are changes, the consequence of which is aging, and in our work we will consider this very period. For this purpose we used the whole genome RNA sequencing data. Currently, the RNA sequencing method has become one of the main ways to study the fundamental mechanisms of aging. In studies on age-related changes in the transcriptome, a decrease in its production during life was found (Kang et al., 2022), as well as multidirectional changes in RNA production levels across individual gene groups (Santra et al., 2019; Meyer and Schumacher, 2021; Stoeger et al., 2022). Summarizing the currently available data on age-associated reduction of gene expression, it can be argued that it contributes to the progressive reduction of cellular functions. Understanding the mechanisms that determine transcriptome aging is necessary to determine the underlying mechanisms of aging (Stegeman and Weake, 2017; Lu et al., 2022; Stoeger et al., 2022). Based on our theoretical model, one of the main questions to test it is whether the level of RNA synthesis and related resources are redistributed between the functional groups of HG and IntG genes during ontogenesis.

In this work we set ourselves the following goal: using mathematical statistics to obtain results capable of verifying our assumptions about the relationship between ontogenesis and aging based on the analysis of the RNA synthesis database. In our work, we encountered limitations related to the database we used. In many ways, such noisy data prevented us from getting statistically significant answers, but we were able to see a number of trends in the data that indirectly support our hypothesis.

## Materials and methods

In this study we used the data and results presented in the articles by the authors (Tabula Muris Consortium, Overall coordination, Logistical coordination, 2018; Ferreira et al., 2021). The RNA-Seq data on the *Mus musculus* mouse genome transcriptome is available in the GEO repository, under the accession number GSE132040 (www.ncbi.nlm.nih.gov/geo/query/acc.cgi?acc=GSE132040). The analyzed database contains the RNA-Seq data from 17 tissue types: *Brain, BAT, Bone, GAT, Heart, Kidney, Lung, Marrow, MAT, Pancreas, SCAT, Skin, Small, Splin, Spleen, WBC, Limb, Liver*. Samples for each tissue type were taken from five or six male and female *Mus musculus* individuals (3M+3F or 3M+2F). For the age dynamics a new group of experimental mice was used each time they reached the age of 1, 3, 6, 9, 12, 15, 18, 21, 24 and 27 months. The *Mus musculus* RNA-seq was converted into FASTQ format and quantified the expression for each gene using Salmon, where GRCm39 (Genome Reference Consortium Mouse Reference 39) was used as a reference transcript for all samples. The tissue types and their labeling are also taken from the database we mentioned earlier. Quality control and initial data normalization were used in the process of extracting them from the database. The quantification output was processed and computed in order to obtain the mean and standard deviation for the gene expression level. Total number of genes from the database was 35,630.

Selection into the HG gene group was performed according to the Housekeeping and Reference Transcript Atlas (HRT Atlas v1.0, (www.housekeeping.unicamp.br) (Hounkpe et al., 2021). Genes responsible for the following cellular functions were included in the group classified as HG: *Transcription factors, Translation factors, RNA splicing, tRNA synthesis, Ribosomal proteins, Mitochondrial ribosomal proteins, RNA polymerase, Protein processing, Histone, Cell cycle, DNA repair/replication, Metabolism, Lysosome, Proteasome, Structural Cytoskeletal, Surface Channels and transporters, Kinases/signalling, Organelle synthesis*. When the HG list was compiled, all variants of genes from those already listed, were additionally included. Total number of the Housekeeping genes was **5,101**. All remaining genes (**30,529**) were assigned to the IntG group. Genes with the same name in both groups were removed from the total list of the whole genome. The stochasticity of the original data due to the use of genetically heterogeneous *Mus musculus* and the small (5 or 6) number of samples to represent the value at each age point impose certain limitations on the use of statistical methods for analyzing the results. These limitations excluded the use of polynomial analysis of variation in the resultant curve or cluster analysis.

Therefore, we applied linear regression analysis to analyze the 27-month behavior of the HG and IntG groups. The F-test was used to compare the slopes of the linear regressions. All *p*-values were based on two-sided hypothesis testing. A *p*-value of less than 0.05 was considered statistically significant. The total gene productivity of all 17 tissue types was considered for each time point. Sometimes the variance of gene productivity between mice in a given experiment was very large. Therefore, time point analysis was performed for genes whose coefficient of variation was <1. The behavior of HG and IntG was examined independently at two intervals from 1 to 9 months and from 9 to 27 months, with the point of separation into the two 9-month age segments chosen *a priori* by us before the analysis began.

## Results

**FIGURE 1:**
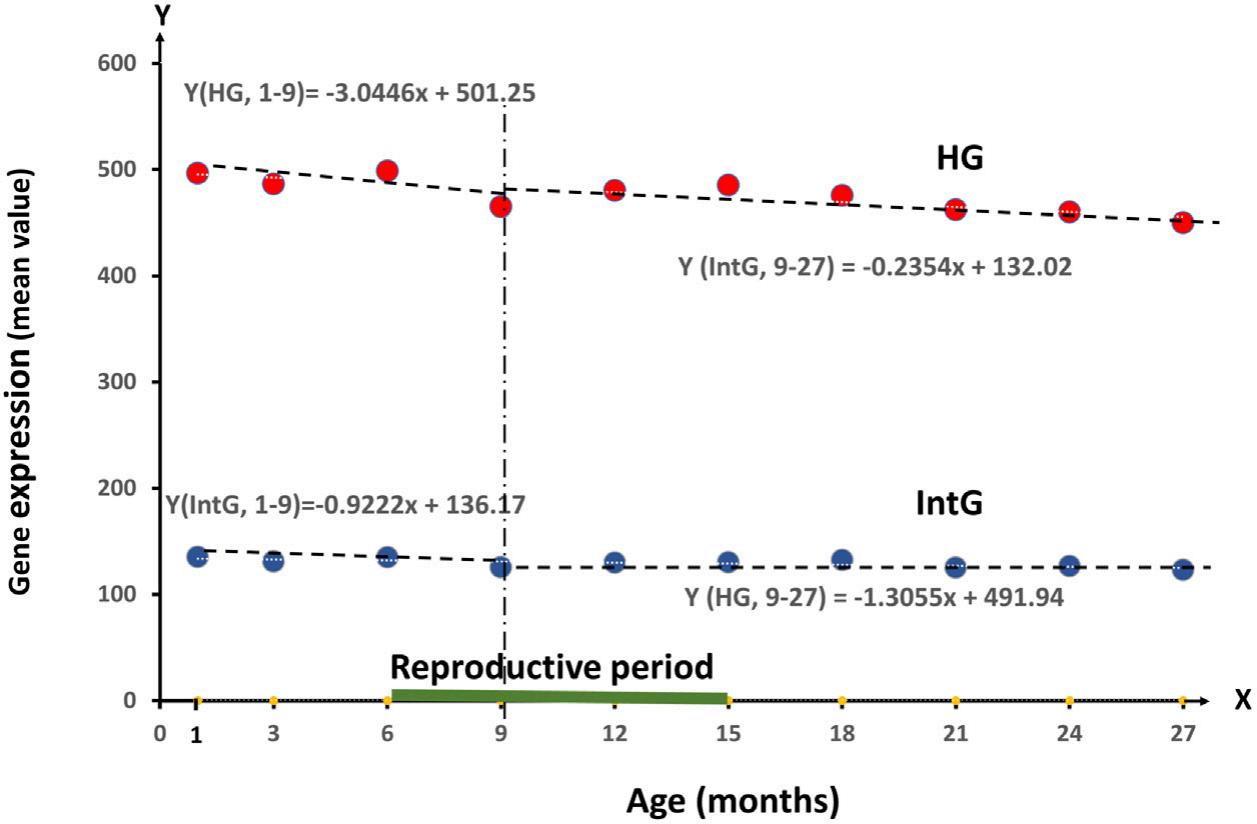
Dynamics of RNA production value of all genes divided into HG and IntG groups in the period from 1 month to 27 months*.*

Giving a general characteristic for the entire database, first of all, we should note a high uneven distribution of the registered activity in the genome. The main amount of production falls on a relatively small group of genes with a high level of RNA synthesis in both HG and IntG groups of genes. Thus, the number of genes that produce more than 80% of all RNA production is only **4,850**, or 13.6% of the total number of genes. At the same time, the genes of the HG group (1,857 genes from total (**4,850)** have slightly higher productivity per gene. One of the main results we obtained in this work is the age-related total dynamics of the studied gene groups. When calculating the combined mean value at each age point for all tissue types, we obtained the statistical data presented in Tab. 1 and Figure 1.

**Table 1:** Total average values (and their confidence intervals) of RNA production in all tissues in the HG and IntG groups during the observation period of age.

Age	Hg means (confidence intervals)	IntG means (confidence intervals)
1	496.39 (95%CI: 474.49–518.29)	135.49 (95%CI: 130.06–140.92)
3	486.62 (95%CI: 464.47–508.78)	130.96 (95%CI: 125.71–136.21)
6	498.84 (95%CI: 476.33–521.35)	134.88 (95%CI: 129.34–140.43)
9	465.32 (95%CI: 444.47–486.18)	125.83 (95%CI: 120.84–130.81)
12	480.44 (95%CI: 459.26–501.62)	130.06 (95%CI: 124.95–135.1)
15	485.38 (95%CI: 462.6–508.16)	130.53 (95%CI: 125.17–135.88)
18	475.54 (95%CI: 454.77–496.3)	132.69 (95%CI: 127.45–137.93)
21	462.22 (95%CI: 441.06–483.38)	125.45 (95%CI: 120.34–130.56)
24	460.17 (95%CI: 439.31–481.02)	126.75 (95%CI: 121.61–131.89)
27	450 (95%CI: 428.02–471.95)	123.14 (95%CI: 118.02–128.27)

The average Gene expression for HG decreases by 3.0446 per month between 1 and 9 months, and by 1.3055 per month between 9 and 27 months, which is statistically significant (*p* = 0.0045). At the same time, IntG dynamics are expressed much less, where there is a decrease in RNA production by 0.9222 per month in the interval 1–9 months and by 0.2354 per month in the interval 9–27 months, giving no statistically significant difference.

The results allow us to conclude that the differences in the level of RNA synthesis between the HG and IntG parts of the genome were statistically highly significant at all age points presented (*p* **-**value < 0.0001). Analyzing the dynamics of changes in the level of RNA production in our isolated HG and IntG parts of the genome, the following results were obtained. For HG as a whole, a decrease per month −1.54 (*t*-test = −4.46, *p*-value = 0.0021) was obtained over the entire time interval of observation, from 1 to 27 months. At the same time for IntG in the same period, a decrease per month was −0.34 (*t*-test = −3.02, *p*-value = **0.0166**). When comparing the values of production decrease between HG and IntG on the whole time interval—from 1 to 27 months using the F test, a value of 10.92 was obtained confirming a statistically significant difference (*p* = 0.0045). Pearson Correlation between Mean HG and Age was −0.84 *p* = 0.0021, and between Mean IntG and Age was −0.73 *p* = 0.0162.

The most interesting for us was to compare the dynamics of HG and IntG in two time intervals: in the period from the beginning of development (1 month) to reaching the peak of physiological development (9 months), as well as from this age to the end of the observation period (27 months).

As can be seen from the linear regression equations, the average Gene expression for HG decreases by 3.0446 per month in the interval 1–9 months, which is statistically significant (*p* = 0.0045) and by 1.3055 per month in the interval 9–27 months. At the same time, the IntG dynamics is expressed much less, where there is a decrease in RNA production by 0.9222 per month in the interval 1–9 months and by 0.2354 per month in the interval 9–27 months, giving no statistically significant difference. Comparing the groups of HG and IntG on the level of regression in both observation periods (1–9) the F test = 2.56 (*p*-value 0.1406) and on 9–27 months F test = 0.86 (*p*-value 0.4067). Statistically significant difference was obtained in both time periods (*p* = 0.0045). The obtained decrease in the level of RNA production in the HG group for all tissues exactly matches the reproductive age in *Mus musculus*, being established in the period from 1 to 9 months and mainly ending by the age of 15 months (Brust et al., 2015). Comparing the data on the *M. musculus* mortality rate with the results obtained, we note that their mortality rate begins to increase significantly from 15 months of age, *exactly when the production of HG genes reaches its minimum value*. Starting from age of 18 months the actual risk of mortality begins to rise, increasing more than twofold to the age of 24 months (Gavrilova and Gavrilov, 2015; Kinzina et al., 2019).

**FIGURE 2:**
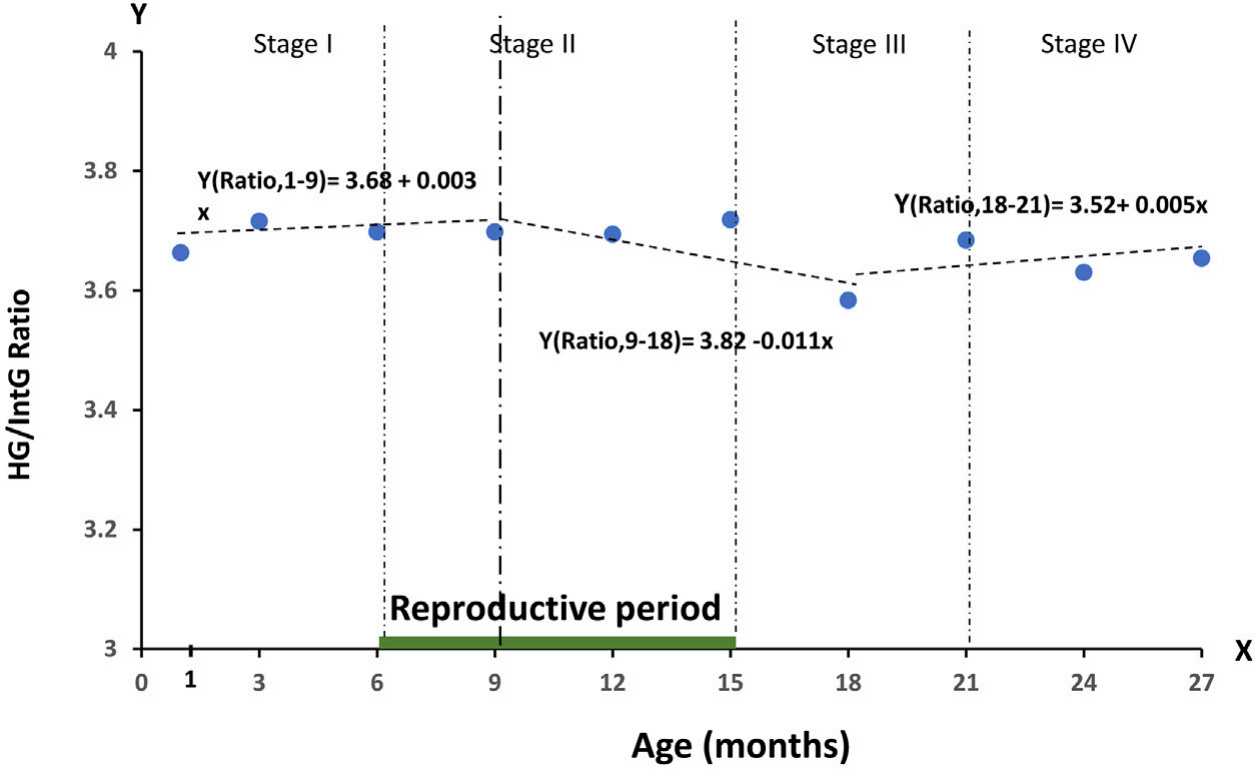
Dynamics of the RNA production ratio—HG/IntG in relative numbers during the observation period of age, divided into four stages of ontogenesis—Stage I (1–6 months), Stage II (6–15 months), Stage III (15–21 months), Stage IV (21–27 months)*.*

Given the considerable scatter of initial data, the most reliable method for comparing the functional groups of genes that we identified is the analysis of their ratio. For this purpose, we obtained data representing the result of HG/IntG ratio in relative figures, presented as a graph in Figure 2.

As can be seen from the graph, the ratio of production of the selected groups of genes HG/IntG changes in different periods of ontogenesis. Thus, in the period of body formation and growth (1–9 months) there is a slight tendency to increase the ratio in favor of HG (the slope value is positive (+0.003 of the average value per month). Further from the end of the reproductive period, when the physical growth of the body is completed, to the middle of the post-reproductive stage of ontogenesis (9–18 months) a significant shift of the ratio in favor of IntG (negative slope −0.011 of the average value per month) is observed with a slight increase at the beginning of old age (21 months). Note that the age point at 21 months corresponds to the age at which the probability of death in mice begins to rise significantly. Thus, the dynamics of HG/IntG gene production ratio shown in Figure 2 clearly demonstrates the relationship between this index and the stages of growth and development regulated by the ontogenesis program. When attempting to analyze the individual tissue types presented in the database, a significant value of the coefficient of variation of the data was found in all individual tissues. Preliminary analysis of the individual tissues revealed a decreasing trend in HG levels from 1 to 15 months in all tissues. The dynamics of the HG/IntG ratio also repeats the behavior of the total ratio. Further, different variations in the activity of this group of genes are observed. Such results are consistent with the data showing heterochronicity of aging processes (Yousefzadeh et al., 2020; Srivastava et al., 2020; Yamamoto et al., 2022). In our future work we plan to study in detail the age-related dynamics of RNA production in various tissues. For this purpose we intend to use in our work databases obtained on experimental animals representing one genetic line (C57BL/6 or BALB/c).

**FIGURE 3:**
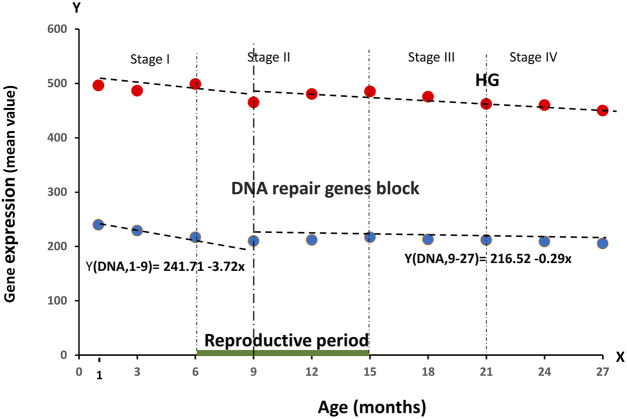
Dynamics of the average RNA production (block of 56 genes) responsible for cellular DNA repair processes during the observation period of age, divided into four stages of ontogenesis—Stage I (1–6 months), Stage II (6–15 months), Stage III (15–21 months), Stage IV (21–27 months)*.*

Of undoubted interest of our work was to determine the overall dynamics of age-related RNA production of genes that perform the necessary function of cellular DNA repair. For this purpose, within the HG group we identified 56 genes responsible for this function according to the list given in the works devoted to this topic (Wood et al., 2005; Knijnenburg et al., 2018). The results are presented in Figure 3.

**FIGURE 4:**
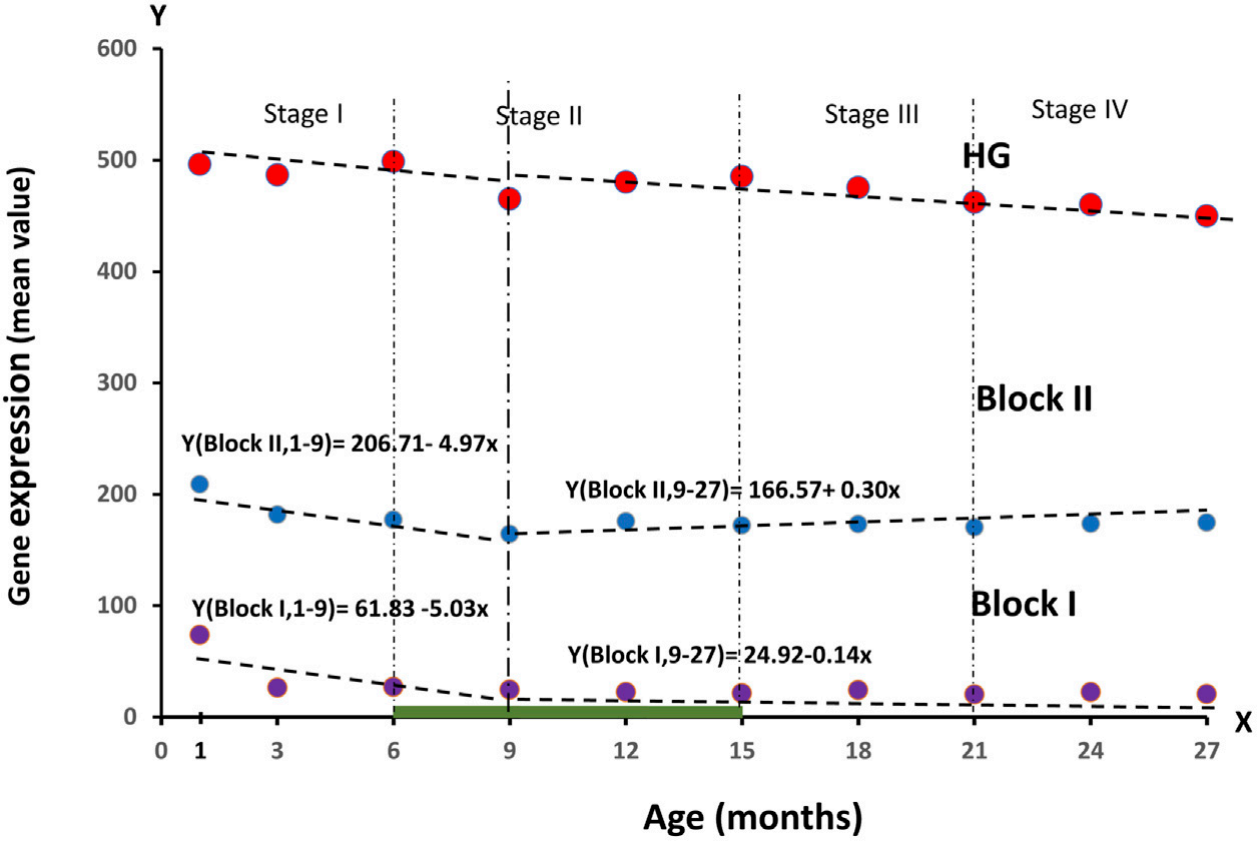
Dynamics of RNA production values in blocks of genes regulating ontogenesis (**Block I**: and **Block II)** during the observation period of age, divided into four stages of ontogenesis—Stage I (1–6 months), Stage II (6–15 months), Stage III (15–21 months), Stage IV (21–27 months).

As can be seen from Figure 3, we obtained a tendency to a smooth decrease in the activity of these genes, repeating the dynamics of the entire HG group. In the age period from 1 to 9 months there was a decrease (241.71-3.72 per month). Subsequently, the value of RNA production of the reparase gene block remained practically unchanged. Such trends in the dynamics indirectly confirm our assumption about the growing infrastructure deficit in the body cells with age. Also one of our main tasks was to compare the age-dependent changes in RNA production of genes responsible for the implementation of the ontogenesis program with the dynamics of HG genes in total. For this purpose, we selected two blocks of such genes, presented in the articles devoted to this topic. Thus**, Block I** contains the main HOXA genes, responsible for the early stages of development and formation of the neuromuscular and skeletal system. **Block II** contains the genes responsible for the formation of the cardiovascular system and the overall level of metabolism (Dou et al., 2016; Peter, 2020). These blocks included the genes are—**Block I**: *HOXA1,HOXA2, HOXA3, HOXA4, HOXA5, HOXA6, HOXA7, HOXA9, HOXA10, HOXA11, HOXA13*; **Block II**: *CDX,CUX1,GDF1,GDF3,GDF5, GDF 10,GDF15,GSX,PAX3, PAX6,SRGAP2,GDF11*. Analysis of the behavior of gene blocks representing the regulation of ontogenesis is shown in Figure 4.

As can be seen from the graph, **Block I** tends to show a significant decrease in values by 3 months. From 1–9 months, the downward trend remained strong (61.83–5.03 drops per month), and remained approximately at the same level from 9 to 27 months. RNA production in **Block II** also declined at 1–9 months (206.71–4.97 declines per month) and little changed thereafter until 27 months of age. Changes in RNA production values in these blocks, especially in **Block I**, precede the decrease in total production in the HG gene group. The relationship between RNA production, ontogenesis stages and mortality risk indicators will be analyzed in the next section of our article.

## Discussion

We agree with the criticism of using the hallmarks of aging as a paradigm, (Gems and de Magalhães, 2021). Our work is aimed at creating the missing new paradigm of understanding and studying the phenomenon of aging. In our opinion, the search for the main mechanism of aging should be aimed at an in-depth study of the work of the leading regulators of ontogenesis. It is in this direction that we plan our future research. For us the fundamental approach is to consider not individual genes, but their ontogenetically determined and various in their role in the organism functional groups necessary both for implementation of the ontogenesis program and for normal functioning of the organism. Again, we proceed from the statement that an insufficient level of repair is the main cause of aging. Understanding exactly how this deficiency occurs is our main task. The connection between ontogenesis and aging is evident to us, in which aging itself is a by-product of the ontogenesis program. Given the limitations associated with the small number of experimental animals used to obtain a result at each time point and the availability of only 10 such points, the results obtained are also limited due to the high noise level in the data.

Let us present our analysis of the results. Putting the obtained data into a whole picture, let us dwell on the comparison of changes in the ratio between the functional groups of HG and IntG genes presented in Figures 1, 2 with the current actual level of mortality risk in *Mus musculus* at different periods of ontogenesis. There is a “resilience zone” with an optimal ratio of minimum mortality, where we observe *a peak of physiological resilience* at 9 months of age (30 years for humans). This age was obtained using a model of growth and distribution of metabolic energy between maintenance of existing tissues and production of new biomass (West et al.(2001), According to this model, the growth curve ends in *Mus musculus* after 9 months (Brust et al., 2015). With the end of the reproductive period and starting from 18 months (60 years), we observed the greatest changes in the HG/Intg ratio. Exactly at this time the probability of mortality begins to increase steadily, increasing more than twice by the age of 24 months (80 years) (Kinzina et al., 2019). Confirmation of changes occurring in the post-reproductive period of ontogenesis (45–50 and 60–65 years) are presented in an article where data were obtained on significant changes in blood plasma composition at these ages (Lehallier et al., 2019). The increasing cascade of changes responsible for aging occurring during this period is accompanied by a constant increase in the probability of death (Kaplan and Robson, 2009), appearing as a side effect of ontogenesis mechanisms. Note that the obtained change of HG/IntG ratio observed at the end of postreproductive and beginning of senile age has its own explanation. For each study of RNA production, a separate group of mice up to a certain age was used. This leads to the fact that only those animals whose peculiarities help them to survive to senile age are included into the group of old mice, probably associated with the possibility to partially restore the optimal HG/IntG ratio. In the course of the ontogenesis program directing the course of differentiation, the target genes get an advantage. A vivid example of such prevalence is skin cells producing keratin or erythrocytes filled with hemoglobin during their maturation. Moreover, in the course of rejuvenation experiments there was shown a direct correlation between the level of cell differentiation and its biological age (Lemaitre, 2014; Brunet et al. 2022). It can be concluded that almost all differentiated cells, except for stem cells, reach their final stage of development already having a disproportion between functional groups of HG and IntG genes, making aging inevitable. The direct connection between ontogenesis and aging program implementation lies in the fact that although this program is aimed at the organism as a whole, it is implemented at the genome level of each individual cell, demonstrating a direct connection between the program and the cell cycle.

In our previous work on genome methylation (Salnikov et al., 2022) we obtained the results confirming significant differences in this parameter between functional groups HG and IntG, which decreases over time. Considering the fact that methylation itself reflects the processes of epigenetic regulation, the results showing the highest level of dispersion of its values in the reproductive age period also deserve attention.

In conclusion, let us separate the established facts from our proposed theoretical justification of our hypothesis. Data analysis showed that the level of RNA production in the HG and IntG genes had statistically significant differences (*p*-value <0.0001) during the entire observation period. During the observation period, statistically significant dynamics of RNA production decrease was determined in the HG group in contrast to the IntG group (*p*-value = 0.0045).

Given the fact that only 10 time points were presented in the database, we were only able to obtain statistically significant results for the slope of the curves (correlation with age) in the population. Less extended time intervals showed only trends. Of these, we highlight the detected significant change in the HG/IntG ratio in the postreproductive stage of ontogenesis indirectly confirms our hypothesis. This imbalance, in turn, leads to insufficient supply of cellular functions with their infrastructure represented in the genome by the HG functional group that may trigger the whole cascade of changes leading to aging.

We are aware of the limitations of the first results of our work, which do not yet provide an opportunity to prove analytically the correctness of our assumptions. However, the first results presented in our work allow us to support the proposed hypothesis, making the chosen direction promising for further research and understanding the mechanisms of aging.

## Data Availability

The original contributions presented in the study are included in the article/supplementary material, further inquiries can be directed to the corresponding author.
